# Supplementary material for: Over Expression of the Cyanobacterial Pgr5-Homologue Leads to Pseudoreversion in a Gene Coding for a Putative Esterase in Synechocystis 6803
Source: Life (Basel). 2020 Sep 3;10(9):174. doi: 10.3390/life10090174 (PMC7555055; doi:10.3390/life10090174)
Supplement: Supplementary file 1 [file life-10-00174-s001.pdf]

life

Supplementary material: Over expression of the cyanobacterial Pgr5-homologue leads to pseudoreversion in a gene coding for a putative esterase in *Synechocystis* 6803

Table S1: PSII parameters from FIRe measurements, σPSII - Effective absorption cross section of PSII in darkness, τ1 PSII – Rate constant for the minimum turnover time of PSII photochemistry after single saturating flash, τ2 PSII – Rate constant for the minimum turnover time of PSII photochemistry after a train of multiple saturating flashes. Error bars represent standard deviation n = 3.

	σ PSII	τ1 PSII	τ2 PSII
WT	121.8 ± 7.5<br>(100%)	102.5 ± 11.5<br>(100%)	1265.3 ± 101.6<br>(100%)
*pgr5*OE	142.0 ± 34.9<br>(117 ± 13%)	90.0 ± 4.0<br>(88 ± 4%)	1321.3 ± 51.1<br>(104 ± 4%)
*pgr5*KO	121.7 ± 9.9<br>(100 ± 8%)	103.7 ± 6.0<br>(101 ± 6%)	1296.0 ± 139.5<br>(102 ± 3%)

Table S2. Western blot quantification of the results in Figure 3. The calculation is based on three repeats (one of the repeats is shown in Figure. 3A.).

	αPsaA	αPsbA
WT<br>-Glu	100% ± 0.0%	100% ± 0.0%
WT<br>+Glu	112.5% ± 43.4%	99.1% ± 7.8%
*pgr5*OE<br>-Glu	126.7% ± 30.6%	121.5% ± 31.2%
*pgr5*OE<br>+Glu	111.7% ± 21.6%	102.9% ± 3.1%

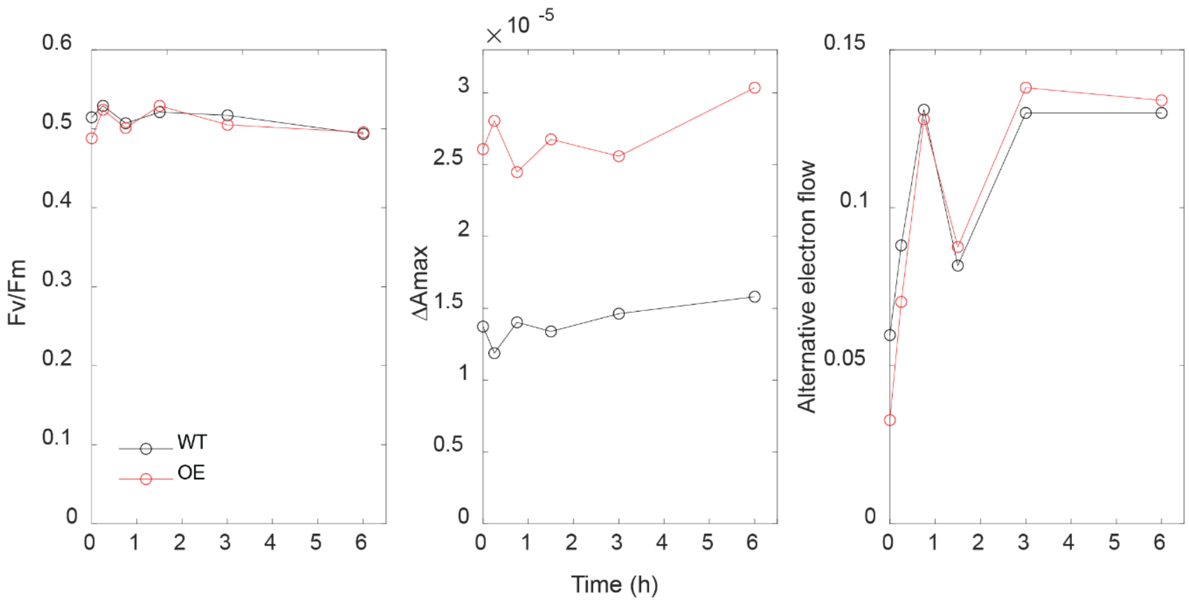

Figure S1. Short-term response to high light after addition of glucose, Cultures were adjusted to OD 730 0.1, then grown on YBG11 medium for 3 days. 5 mM glucose was added at T0. Samples were immediately moved from 40 to 600 μmol photons m^{-2} s^{-1}. (A) Photochemical efficiency (Fv/Fm) of photosystem. (B) Maximal PSI activity, ΔAmax, normalized to number of cells. (C) Fraction of alternative electron flow, calculated as the fraction of the area between the DCMU and DCMU & DBMIB measurements (supplementary Figure S3).

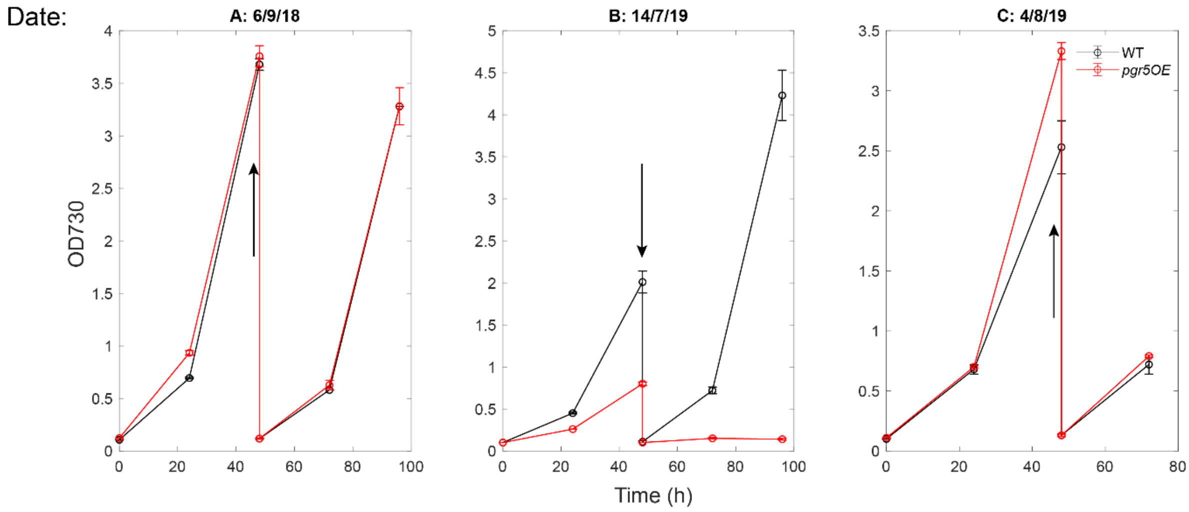

Figure S2. Biomass accumulation over time, Cultures were grown on YBG11 medium with 5 mM glucose for five days. Biomass was monitored as optical density at 730 nm. Error bars represent standard deviation derived from three repeats. The arrow marks the point at which the cultures where diluted. (A) 6.9.18 - Representative growth curve of the *pgr5OE* mutant after the loss of the collapsing phenotype. This *pgr5*OE harbors slr1916 point mutation (B) 14.7.19 - Cultures from the original mutation were taken out of glycerol stock collapse in the presents of glucose in the media. (C) 4.8.19 – cultures from glycerol stock stop collapsing.

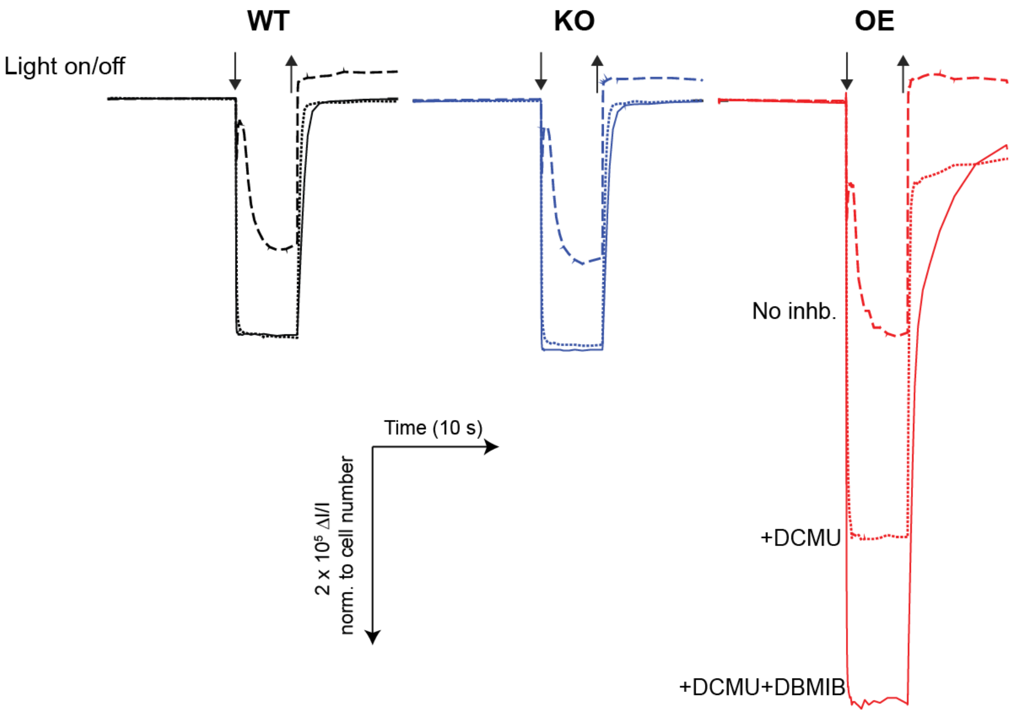

Figure S3. P700 photochemical activity calculations, Absorbance changes were measured in vivo in cultures grown in YBG11 medium with 5 mM glucose. The data shown is normalized to the number of cells. P700 oxidation was measured as the absorbance changes at 705 nm using 590 μmol photons m-2 s-1 actinic light intensities. Arrows indicate the illumination period. Dashed lines - no inhibitors; dotted lines following the addition of DCMU;, solid lines after the addition of DCMU and DBMIB. The contribution of alternative sources to PSI electron flow was calculated as the fraction of the area trapped between the +DCMU and +DCMU+DBMIB curves, normalized to the +DCMU+DBMIB measurement:

$$\frac{\int_{light\ off}^{Light\ on}(+DCMU + DBMIB) - \int_{light\ off}^{Light\ on}(+DCMU)}{\int_{light\ off}^{Light\ on}(+DCMU + DBMIB)}$$

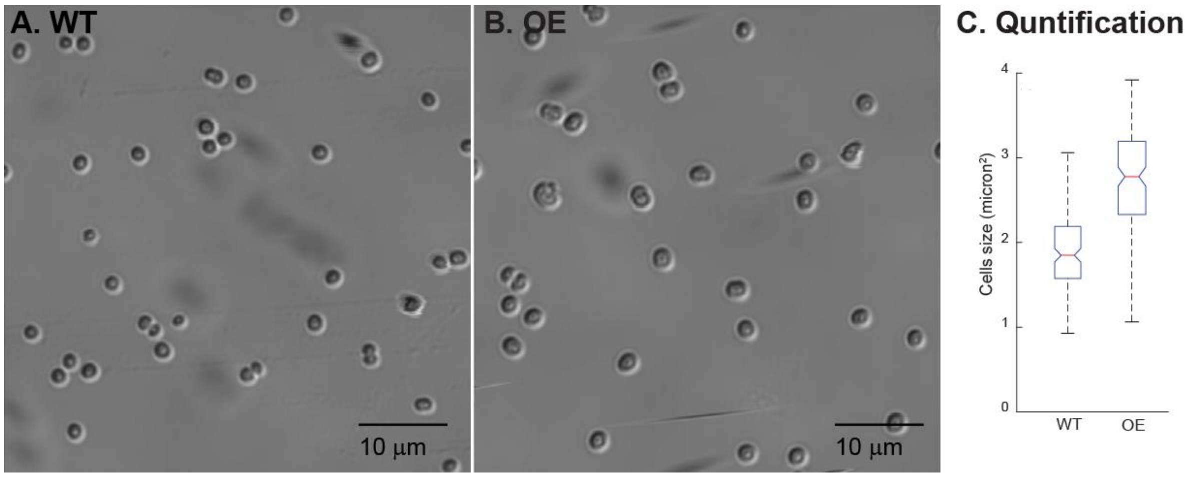

Figure S4. Cell size. Representative confocal images of cultures. (A) WT and (B) *pgr5*OE. The images presented here are from cultures grown for 5 days in YBG11 media. (C) Distribution of cell sizes measured by confocal microscopy. In this box plot the central mark indicates the median, the bottom and top edges of the box indicate the 25th and 75th percentiles.

© 2020 by the authors. Submitted for possible open access publication under the terms and conditions of the Creative Commons Attribution (CC BY) license (http://creativecommons.org/licenses/by/4.0/).
